# Synthesis and Characterization of Manganese Dithiocarbamate Complexes: New Evidence of Dioxygen Activation

**DOI:** 10.3390/molecules26195954

**Published:** 2021-09-30

**Authors:** Petra Martini, Alessandra Boschi, Lorenza Marvelli, Licia Uccelli, Stefano Carli, Giuseppe Cruciani, Erika Marzola, Anna Fantinati, Juan Esposito, Adriano Duatti

**Affiliations:** 1 Department of Translational Medicine, University of Ferrara, Via Fossato di Mortara, 70 c/o Viale Eliporto, 44121 Ferrara, Italy; petra.martini@unife.it (P.M.); licia.uccelli@unife.it (L.U.); 2 Department of Chemical, Pharmaceutical and Agricultural Sciences, University of Ferrara, Via L. Borsari, 46, 44121 Ferrara, Italy; lorenza.marvelli@unife.it (L.M.); stefano.carli@unife.it (S.C.); erika.marzola@unife.it (E.M.); anna.fantinati@unife.it (A.F.); dta@unife.it (A.D.); 3 Department of Physics and Earth Sciences, University of Ferrara, Via Saragat, 1, 44122 Ferrara, Italy; giuseppe.cruciani@unife.it; 4 Legnaro National Laboratories, Italian National Institute for Nuclear Physics (LNL-INFN), Viale dell’Università, 2, 35020 Legnaro, Italy; juan.esposito@lnl.infn.it

**Keywords:** manganese complexes, diethyldithiocarbamate, manganese(III) complexes, dioxygen activator

## Abstract

(1) Background: Metal dithiocarbamate compounds have long been the subject of research due to their ease of formation, excellent properties and potential applications. However, manganese complexes with dithiocarbamates, to our knowledge, have never been used for medical imaging applications. With the aim of developing a new class of mononuclear manganese(II)-based agents for molecular imaging applications, we performed a specific investigation into the synthesis of mononuclear bis-substituted Mn(II) complexes with dithiocarbamate ligands. (2) Methods: Synthesis in either open or inert atmosphere at different Mn(II) to diethyldithiocarbamate molar ratios were performed and the products characterized by IR, EA, ESI-MS and XRD analysis. (3) Results: We found that only under oxygen-free atmospheric conditions the Mn(II) complex MnL_2_, where L = diethyldithiocarbamate ligand, is obtained, which was further observed to react with dioxygen in the solid state to form the intermediate superoxo Mn(III) complex [MnL_2_(η^2^-O_2_)]. The existence of the superoxo complex was revealed by mass spectroscopy, and this species was interpreted as an intermediate step in the reaction that led the bis-substituted Mn(II) complex, MnL_2_, to transform into the tris-substituted Mn(III) complex, MnL_3_. A similar result was found with the ligand L’ (= bis(N-ethoxyethyl)dithiocarbamate). (4) Conclusions: We found that in open atmosphere and in aqueous solution, only manganese(III) diethyldithiocarbamate complexes can be prepared. We report here a new example of a small-molecule Mn(II) complex that efficiently activates dioxygen in the solid state through the formation of an intermediate superoxide adduct.

## 1. Introduction

Dithiocarbamates (-S_2_CNR_2_, R = general organic functional group) are highly versatile chelating ligands, forming stable complexes with both transition and main group elements. Transition metal complexes with this class of ligands have long been the object of research due to their easy formation, excellent properties and potential applications [1,2]. In terms of group VII transition metals, it is worth noting that, while mononuclear technetium-99m and rhenium-188 dithiocarbamate radio-compounds are used in the field of nuclear medicine as imaging and therapeutic agents, respectively [3,4], manganese complexes with dithiocarbamates, to our knowledge, have never been used for medical applications. A possible reason could be the chemical nature of these complexes, prepared from Mn(II) salts that still remain a matter of debate.

This work is part of the project METRICS (acronym for Multimodal pET/mRi Imaging with Cyclotron-produced ^51/52^Mn iSotopes), which aims to develop the technology needed to obtain a cyclotron-driven production of ^52/51^Mn PET radionuclides and to achieve a perfect molecular matching by taking advantage of both paramagnetic and radioactive properties shown by manganese isotopes in order to enable PET/MRI hybrid imaging. In this framework, we started an investigation into the class of dithiocarbamate ligands with the purpose of developing a new class of mononuclear manganese(II)-based agents for medical applications. In particular, we performed specific studies on the synthesis of mononuclear bis-substituted Mn(II) complexes with dithiocarbamate ligands. It is known that several studies on manganese diethyldithiocarbamate complexes prepared from Mn(II) salts have been performed and reported in the literature; however, the chemical nature of these compounds and their stability in open atmosphere still remain a matter of debate. Some studies demonstrated, in particular, that the reactivity between the Mn(II) ion with the diethyldithiocarbamate ligand in aqueous solution to form manganese bis-dithiocarbamate complexes seems to be particularly related to the presence or absence of dioxygen, while not completely clarifying the species that are formed in different reaction conditions [5,6,7,8,9,10,11,12]. With the purpose of determining the nature of these species, we reported in this work the synthesis and the characterization of Mn(III) and Mn(II) dithiocarbamate complexes that are formed in open or oxygen-free atmospheric conditions using different metal–ligand molar ratios. In particular, we found that the mononuclear complex [Mn(II)(S_2_CNEt_2_)_2_] (abbreviated MnL_2_, where L = diethyldithiocarbamate anion, [S_2_CNEt_2_]^−^ (IUPAC name: diethylcarbamodithioate)) could only be prepared under strictly nitrogen-controlled conditions; and, surprisingly, we discovered that this species is an efficient O_2_-activating complex, although this relevant property has gone largely unnoticed. Similar results were found with the ligand L’ (= bis(N-ethoxyethyl)dithiocarbamate (IUPAC name: L bis(2-ethoxyethyl)carbamodithioate)). The study led to the isolation and spectroscopic characterization of intermediate Mn–superoxo complexes that were not detected previously.

## 2. Results and Discussion

### 2.1. Synthesis and Characterization of MnL_2_ (***1***), (L = Diethyldithiocarbamate)

The synthesis of **1** was performed under inert conditions by reacting the Mn(II)Cl_2_•4H_2_O complex with two equivalents of the sodium salts of the dithiocarbamate ligand L (Na[(CH_3_CH_2_)_2_-N-C(=S)S]•3H_2_O), after purging of all the reaction containers and water solvent by nitrogen bubbling and by the Schlenk technique. It is important to note that dropwise addition of the solution of MnCl_2_ is necessary to prevent the possible formation of bridged polymetallic species. The reaction immediately led to the formation of a yellow precipitate, isolated in good yield. Always keeping the environment under nitrogen flow, the yellow precipitate was filtered and washed with degassed water. When kept under strictly anhydrous conditions under an inert nitrogen atmosphere, the yellow solid product was indefinitely stable.

The compound **1** is soluble in dichloromethane, chloroform and acetonitrile. The infrared spectra of the ligand L exhibit characteristic bands at 1476 cm^−1^, assigned to ν(C–N) (thioureide) stretching vibrations. These bands are shifted to a higher frequency in the product **1** (1497 cm^−1^), presumably due to the increase in C–N double bond character as a consequence of the effective involvement of the nitrogen lone pair in resonance upon coordination of the diethylthiocarbamate ligand to the manganese ion [12] (Supplementary Figure S1). This indicates that the C–N bond order lies between (C–N) single (1250–1350 cm^−1^) and (C=N) double bond (1640–1690 cm^−1^) [13]. Similarly, a shift is observed in the single band due to ν(C–S) stretching vibration from 986 cm^−1^, in the ligand, to 990 cm^−1^ upon coordination. At these frequencies, the presence of a single peak indicates that the ligand bonds to the metal center in a symmetrical bidentate chelating mode [14,15]. The absence of the absorption band in the region 3400–3650 cm^−1^, due to the stretching frequency of the functional group –OH, excludes the coordination of water molecules or hydroxyl groups to the metal. This band is clearly visible in the IR spectrum of Na[(CH_3_CH_2_)_2_-N-C(=S)S]•3H_2_O.

The [ESI(+)-MS] analysis of samples collected immediately after preparation (Supplementary Figure S2) indicated the presence of a main peak with *m*/*z* of 351.07 corresponding to the species MnL_2_.

### 2.2. Stability Studies of MnL_2_ (***1***) after Exposure to Atmospheric Dioxygen

**Figure 1:**
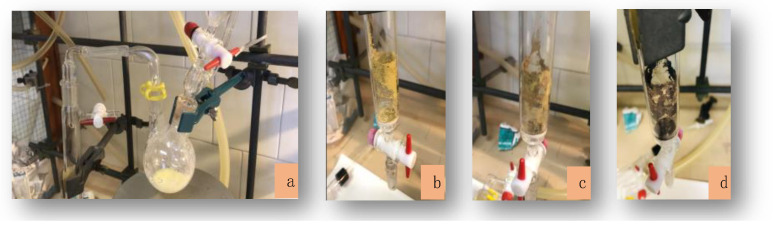
Pictures of the synthesis of MnL_2_ under inert conditions: (**a**) the yellow precipitate that forms under inert atmosphere; (**b**) the yellow precipitate after filtration and washing under inert atmosphere; (**c**) color darkening when it comes into contact with air; (**d**) brown product after 60 min contact with air.

When the solid yellow product **1** comes into contact with air, it changes color, becoming brown-yellow at first and then turning dark brown (Figure 1). This transformation can be slowed down only by maintaining controlled atmospheric working conditions.

The XRD diffraction pattern (Supplementary Figure S3) obtained immediately after the formation of the newly brown sample showed the presence of the bis-substituted complex MnL_2_ (**1**), previously characterized by Fackler and colleagues [10], and an unidentified phase. The brown product was analyzed by ESI mass spectroscopy in positive ion mode [ESI(+)-MS]. While the [ESI(+)-MS] spectra of samples collected immediately after the opening of the air flow are dominated by the presence of a main peak at *m*/*z* = 351.1 corresponding to the bis-substituted [MnL_2_] complex (Supplementary Figure S2), the [ESI(+)-MS] spectra of samples collected at later times reveal some remarkable features. The spectrum (Supplementary Figure S4) is characterized by the presence of two main peaks at *m*/*z* = 351.15 and *m*/*z* = 383.06. A smaller peak is observed at *m*/*z* = 499.50. The peak at *m*/*z* = 383.06 is consistent with the formation of the Mn(III) superoxide adduct [MnL_2_(η^2^-O_2_)] (compound **2**) that can be generated from **1** by O_2_ one-electron oxidation of the Mn(II) metallic center [7,16,17,18,19,20]. Attempts at peak assignment suggest also that the peaks at *m*/*z* = 319.31 and 303.2 are consistent with the species [MnLH_2_O_2_(CH_3_CN)_2_]^+^ and [MnLH_2_O(CH_3_CN)_2_]^+^, respectively, generated in CH_3_CN after the loss of the ligand L during mass analysis [17]. The peak found at *m*/*z* = 351.15 in the [ESI(+)-MS] spectrum is postulated to correspond to the Mn(III) fragment [MnL_2_]^+^ generated by the leakage of the superoxo ligand O_2_^−^. Finally, the peak at *m*/*z* = 499.50 is easily interpreted as corresponding to the production of the tris-substituted Mn(III) complex [MnL_3_].

Elemental analyses of the dark product, collected 24 h after exposure to atmospheric oxygen, were in satisfactory agreement with theoretical values predicted for the formulation [MnL_2_(η^2^-O_2_)], where the O_2_ ligand is supposedly coordinated in a side-on mode to preserve the octahedral arrangement. The electronic spectrum (ES) of the dark product (Figure S5), collected in the region 800–200 nm in chloroform, was also in satisfactory agreement with the hypothesis of the octahedral arrangement. The ES is dominated by an intense band in the 235–300 nm UV range attributed to intra-ligand transitions and a lower intensity band at 355 nm probably due to ligand–metal transitions. In the Vis region, we observed a weak band at 498 nm and a shoulder at 606 nm, which could only be analyzed with the concentrated sample, attributed to d–d transitions compatible with the assumed configuration of pseudo-octahedral complexes [21,22].

This side-on binding type of the superoxide ligands was further supported by the presence of new bands at 969.32 cm^−1^ and 818 cm^−1^ in the FT IR spectrum, absent in the FT IR spectrum of **1** [16] (Supplementary Figure S6).

**Figure 2:**
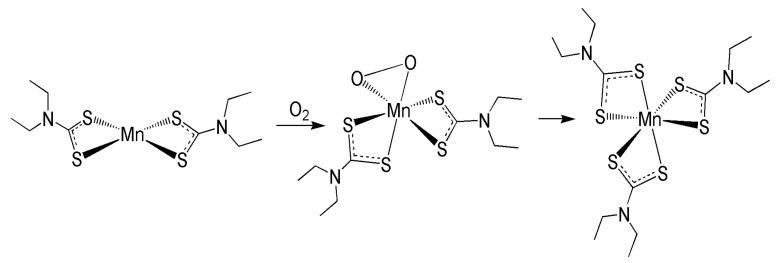
Hypothetical scheme of the conversion of [MnL_2_] into [MnL_3_].

These observations can be nicely explained by assuming the simple reaction mechanism illustrated in Figure 2. In the first step, activation of atmospheric O_2_ by the solid complex [MnL_2_] generates the superoxide intermediate [MnL_2_(η^2^-O_2_)] that is subsequently slowly converted into the tris-substituted complex [MnL_3_] after release of oxygen. The conversion of yellow [MnL_2_] to dark violet [ML_3_] occurred rapidly when the yellow compound was dissolved in chloroform under open air or treated with Et_2_O and, in general, with organic solvents.

The stability of the solid product **2**, stored with free access of air, was monitored over time by acquiring FT IR and XRD spectra of the sample. After 10 days, the color of the sample became brown-ocher, and the FT IR (Supplementary Figure S7) spectrum collected at this time shows a new band at 3439 cm^−1^, compatible with the decomposition hypothesis of the product to hydroxo species. The XRD analysis of the same sample (Supplementary Figure S8) shows the presence of the hausmannite species Mn_3_O_4_ and of N,N-diethyl[(diethylcarbamothioyl)disulfanyl]carbothioamide (C_10_H_20_N_2_S_4_, disulfiram), confirming the reaction of oxygen with the manganese complex **1** that decomposes over time. It is noted that the signals corresponding to the compound MnL_2_ (**1**) in the XRD pattern completely disappeared.

### 2.3. Synthesis and Characterization of [MnL_3_] (3), (L = Diethyldithiocarbamate)

**Figure 3:**
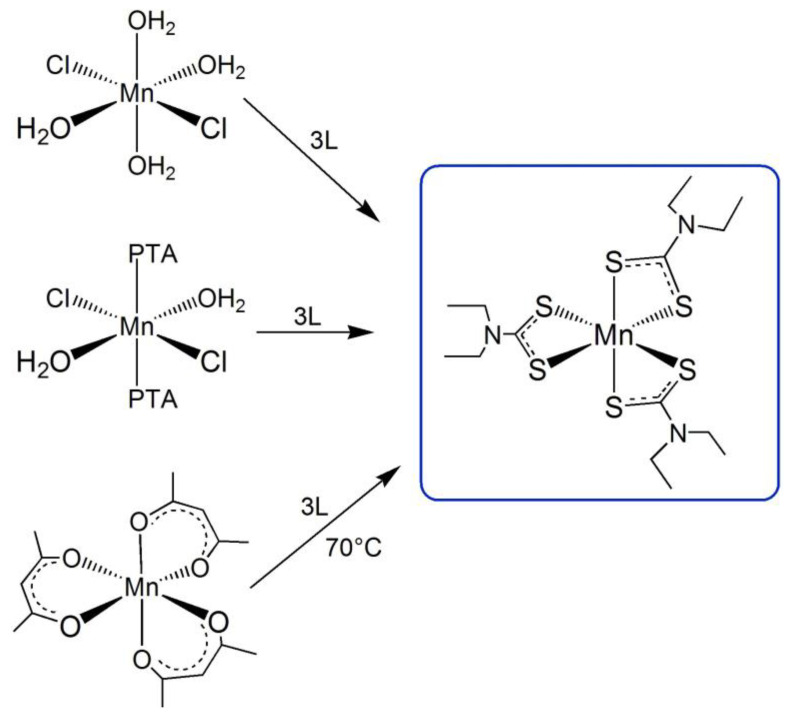
Scheme of the different routes investigated for the formation of the [MnL_3_] (**3**).

In Figure 3, the different routes investigated for the formation of the [MnL_3_] (***3***) compound are schematized.

The reaction of MnCl_2_ with three equivalents of L, in aqueous solution under open atmosphere, led rapidly to the formation of a dark violet solution. After extraction with chloroform and evaporation of the solvent, a dark violet residue was obtained and washed with water and ethanol. Characterization of the violet solid revealed that it corresponded to the tris-substituted Mn(III) complex MnL_3_ (**3**) [23]. The ESI(+)-MS spectrum was characterized by the presence of a main peak at *m*/*z* = 499.40 (Supplementary Figure S9).

The electronic spectrum of **3** (Supplementary Figure S10), collected in the region 800–200 nm in chloroform, is dominated by intense bands in the 235–300 nm UV range attributed to intra-ligand transitions and by a lower intensity band at 353 nm probably due to ligand–metal transitions. The Vis region shows a weak band at 464 nm, which could only be determined by analyzing a concentrated sample, attributed to d–d transitions compatible with a d^4^ configuration of octahedral complexes of Mn(III) as reported in the literature [21,24,25,26]. The cyclic voltammetry carried out in a dichloromethane-based electrolyte, in agreement with AR Hendrickson et al. [27], showed a quasi-reversible redox process (ratio of anodic and cathodic peak currents I_p-ox_/I_p-red_ = 1.1; separation peaks ΔE = 140 mV) centered at +0.25 V vs. NHE relative to the pair Mn(III)/Mn(IV) (Supplementary Figure S11).

The tri-substituted MnL_3_ complex could also be obtained by the reaction of the complex [Mn(II)(PTA)(Cl)_2_(H_2_O)_2_] with the ligand L at room temperature in open atmosphere. In particular, a clear, colorless solution containing the Mn(II) compound was slowly added to an aqueous solution containing 3 equivalents of the ligand L. EA, IR and MS analysis conducted on the product (spectra not reported here) are superimposable on the analysis conducted on the product obtained starting from MnCl_2_, confirming that also this reaction leads to the formation of the [Mn(III)(L)_3_] species. 

With the aim of confirming the formation of **3** obtained from the reaction of the Mn(II) ion with the ligand L, further investigation was carried out by reacting the Mn(III) compound Mn(III)acetylacetonate [Mn(acac)_3_] with L at 70 °C. The reaction was carried out at high temperature in order to favor the exchange reaction of the ligands. The dark violet reaction precipitate was then separated from the environment of reaction and washed with EtOH and H_2_O. EA and IR analysis conducted on the product confirmed the formation of the tri-substituted MnL_3_ species. The analysis of a solution containing the complex by ESI(+)-MS showed again the molecular ion of the oxidized complex with *m*/*z* of 499, corresponding to the species [Mn(L)_3_]^+^.

### 2.4. Synthesis and Characterization of [Mn(II)((S_2_CN(CH_2_CH_2_OEt)_2_)_2_] (***4***), L’ = bis(N-Ethoxyethyl)Dithiocarbamate and Stability Studies after Exposure to Atmospheric Dioxygen

Similar studies performed on the complex [Mn(II)((S_2_CN(CH_2_CH_2_OEt)_2_)_2_] (**4**), abbreviated MnL’_2_ (where L’ = bis(N-ethoxyethyl)dithiocarbamate anion, [S_2_CN(CH_2_CH_2_OEt)_2_]^−^), confirm the reaction of the Mn(II) dithiocarbamate complex with atmospheric dioxygen. Compound **4** was prepared by addition of the compound MnCl_2_·4H_2_O to an aqueous solution containing two equivalents of the sodium salt of ligand L’ (NaL’), after careful purging of all the reaction containers and water solvent by nitrogen bubbling and by the Schlenk technique. The reaction immediately led to the formation of a yellow precipitate, separated in good yield in oxygen-free conditions. Always keeping the environment under nitrogen flow, the yellow precipitate was filtered and washed with degassed water. The infrared spectrum of the compound **4** (Supplementary Figure S12) shows characteristic bands due to the ν(C–N) stretching vibration at 1473 cm^−1^ and a single band due to ν(C–S) stretching vibration at 997 cm^−1^, which indicates that the ligand bonds to the metal center in a symmetrical bidentate chelating mode [5,14,15].

After exposure of the product **4** to atmospheric dioxygen, the yellow powder started to darken and progressively turned into a dark brown solid, similar to what was observed with the product **1**. Analyses of the dark brown solid product are consistent with the formation of the Mn(III) superoxide adduct [MnL’_2_(η^2^-O_2_)] (**5**) that can be generated from **4** by O_2_ one-electron oxidation of the Mn(II) metallic center [17,18,19,20,21,22]. Elemental analyses of the dark brown product, collected 24 h after exposure to atmospheric oxygen, were in satisfactory agreement with theoretical values predicted for the formulation [MnL’_2_(η^2^-O_2_)], where the O_2_ ligand is supposedly coordinated in a side-on mode to preserve the octahedral arrangement. This side-on binding type of the superoxide ligands is further supported by the presence of a new band at 976.62 cm^−1^ in the FT IR spectrum of the dark brown product (Supplementary Figure S13). The peak found at *m*/*z* = 323.59 in the [ESI(+)-MS] spectrum (SM, Figure S14) was postulated to correspond to the Mn(III) fragment [MnL’O_2_]^+^ generated by the leakage of one L’ ligand of the [MnL’_2_(η^2^-O_2_)] compound. Finally, the presence of a smaller peak at *m*/*z* = 763.63 was interpreted as corresponding to the production of the tris-substituted Mn(III) complex MnL’_3_. When the dark brown product was treated with 5 mL of ether, an insoluble yellow-ocher precipitate was separated from the organic brown solution. The FT IR spectrum of the yellow-ocher powder is consistent (SM, Figure S15) with the FT IR spectrum of hausmannite. After solvent evaporation of the brown organic solution, a dark violet residue was obtained and washed with water and ethanol. Characterization of the violet solid revealed that it corresponded to the tris-substituted Mn(III) complex MnL’_3_ (**6**) (Supplementary Figure S16).

We also observed that when the dark brown product was stored with free access of air, the solid changed from dark brown to ocher.

### 2.5. Magnetic Susceptibility

Effective magnetic moments (μ_eff_) of **2**, **3**, **5** and **6** were obtained using the Evans NMR method, with the complexes dissolved in a mixture of CDCl_3_ and CHCl_3_ [28]. 

The effective magnetic moment was calculated from the Equation (2) in Bohr magneton units (μB) by noting the chemical shift difference of CHCl_3_ in the presence and absence of the Mn complex (Supplementary Figure S17). Room temperature magnetic moments of 2, 3, 5 and 6 (Table 1) lie within the range of 4.3 to 4.5 μB, in agreement with the calculated value of 4.90 μB for a d^4^ high spin ion and characteristic of high spin octahedral Mn(III) complexes [29]. The obtained values are in accordance with the [Mn(acac)_3_] experimental value determined by the same NMR procedure for comparison.

The effective magnetic moment determinations of **1** and **4** were not reliable, due to the rapid transformation of the yellow compounds into dark brown in CDCl_3_ and CHCl_3_, and, for this reason, are not reported.

### 2.6. Remarks

When an aqueous solution of the compound MnCl_2_·4H_2_O was added dropwise to another aqueous solution containing two equivalents of the sodium salt of ligand L and L’ in water, and under strictly nitrogen-controlled conditions, the yellow bis-substituted complexes (**1** and **4**) were suddenly formed in high yield.

When the complexes **1** and **4** came into contact with air, the yellow powder started to darken and progressively turned into a dark brown solid. Analyses of the products are consistent with the formation of the Mn(III) superoxide adduct (**2** and **5**) that can be generated from **1** and **4** by O_2_ one-electron oxidation of the Mn(II) metallic center [7,16,17,18,19,20]. The effective magnetic moment results are in agreement with a d^4^ high spin configuration characteristic of an octahedral Mn(III) environment. Elemental analyses of the dark products, collected 24 h after exposure to atmospheric oxygen, of **1** and **4** were in satisfactory agreement with theoretical values predicted for the formulation [MnL_2_(η^2^-O_2_)], where the O_2_ ligand is supposedly coordinated in a side-on mode to preserve the octahedral arrangement. The course of the reaction between solid compound MnL_2_ (**1**) and atmospheric dioxygen was monitored over time, and it was found that the ultimate product was always the mixed Mn(II)/Mn(III) oxide hausmannite and disulfiram (C_10_H_20_N_2_S). Therefore, the occurrence might be speculated of an oxo reduction mechanism that reduces Mn(III) to Mn(II) and oxidates the diethylcarbamodithioate ligand to disulfiram.

**Figure 4:**
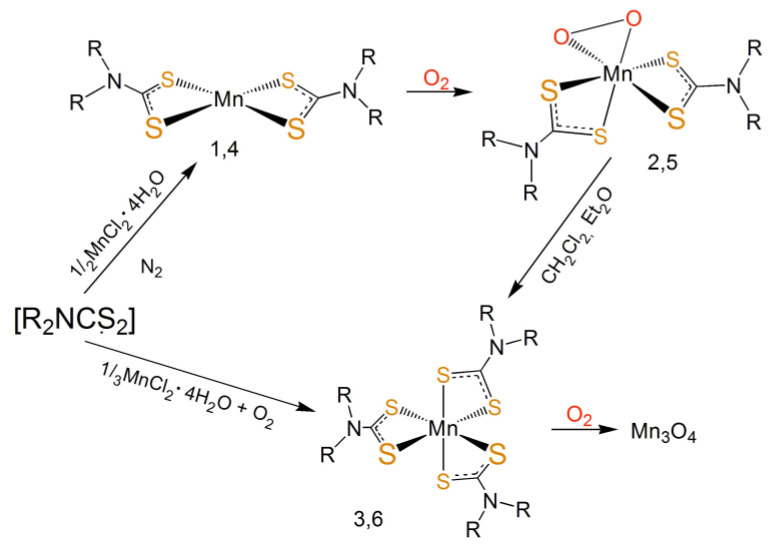
Proposed reaction schemes of Mn(II) and the ligands L and L’ in dioxygen-free atmospheric conditions and in open atmosphere. Compounds **1**,**2**,**3**: R = -CH_2_CH_3_ (L); compounds **4**,**5**,**6**: R = -CH_2_CH_2_OCH_2_CH_3_ (L’).

The reaction of Mn(II) with three equivalents of the dithiocarbamate, in aqueous solution and under open atmosphere, led rapidly to the formation of the dark violet tris-substituted Mn(III) complexes MnL_3_ (**3**) and MnL’_3_ (**6**) (Figure 4). The conversion of yellow [MnL_2_] to dark violet [MnL_3_] occurred rapidly when the yellow compound was treated under open air with organic solvents.

## 3. Materials and Methods

Na[(CH_3_CH_2_)_2_-N-C(=S)S]x3H_2_O and Na[S_2_CN(CH_2_CH_2_OEt)_2_] were obtained from Aldrich Chimica and Alchemy, respectively. Manganese(III) acetylacetonate was obtained from Sigma-Aldrich.

The heterocyclic phosphine 1,3,5-triaza-7-phosphaadamantane (PTA) and the complex [Mn(II)(PTA)(Cl)_2_(H_2_O)_2_] were prepared according to the procedure reported in the literature [30].

The UV–Vis spectra were recorded with a Cary Series UV–Vis Spectrophotometer instrument (model G9823A) in the range 800–200 nm.

The EA analyses were performed by means of the EA FLASH 2000 Thermofisher. MS-ESI analyses were recorded with a Waters ESI Micromass ZQ2000, dissolving the samples in CH_3_CN. 

The infrared spectra (IR) were recorded with a FT-VERTEX 70 in the range 4000–400 cm^−1^ using anhydrous KBr.

Cyclic voltammetry was carried out using a standard three-electrode cell with a Autolab PGSTAT 302/N potentiostat at a scanning rate of 50 mV s^−1^ with a pseudo-reference Ag wire, a Pt wire as an auxiliary and glass carbon as the working electrode. A ferrocene (F_c_)/ferricinium (F_c_^+^) redox couple was used as the internal standard (E^0^ = +0.400 V vs. NHE) [31]. A solution of 0.1 M TBACl in chloroform was used as inert electrolyte.

XRD analyses were performed using the X-ray diffractometer D8 ADVANCE with DAVINCI Design from Bruker AXS.

The effective magnetic moments (*μ_eff_*) of the compounds **2**, **3**, **5** and **6** were determined at 296.3 K using the Evans NMR method [29] and a 400 MHz Agilent Mercury NMR spectrometer. Typically, 2 mg of the compound, dissolved in 0.6 mL of a mixture of deuterated:proteo chloroform (50:1 *v*/*v*), were placed in a NMR tube along with a capillary containing the same solvent mixture.

By recording the chemical shift difference of the solvent molecule in the presence and absence of a paramagnetic species, the molar magnetic susceptibility ($X_{\text{m}}$) can be obtained via Equation (1):

(1) $$X_{\text{m}}=\frac{3\Delta f}{4\pi Fc}\;$$

where Δf is the frequency difference in Hz between the shifted resonance and the pure solvent resonance, F is the spectrometer radiofrequency in Hz, and c the concentration of paramagnetic species (mol/mL).

The magnetic moment is then calculated using Equation (2):

(2) $$\mu_{eff}=\sqrt{8\left(X\text{m}\;T\right)}$$

where *T* is the temperature in Kelvin.

The effective magnetic moment of the commercial Mn(III)acetylacetonate [Mn(III)(acac)_3_] was also measured in the same mixture solution and used as Mn(III) complex standard for the validation of the experimental method.

### 3.1. Synthesis and Characterization of MnL_2_ (**1**)

The compound **1** was prepared by dropwise addition of MnCl_2_•4H_2_O (1.26 mmol, 0.250 g) in 3 mL Milli-Q water to two equivalents of the sodium salt of the ligand L (NaL•3H_2_O) (2.70 mmol, 0.607 g) previously dissolved in 7 mL of Milli-Q water. The preparation was carried out under oxygen-free conditions. The immediate formation of a yellow precipitate was observed which, after leaving the reaction for 30 min under magnetic stirring, was filtered and washed with water, still in a controlled atmosphere. The isolated precipitate (0.314 g), if kept in a controlled atmosphere, maintains a yellow color. (Yield, 71%.) The compound **1** is soluble in acetonitrile, chloroform and acetone. ESI-MS: [Mn(S_2_CNEt_2_)_2_], expected *m*/*z* for C_10_H_20_NS_2_Mn = 351.00; found *m*/*z* = 351.07 [M]^+^. FT IR: ν(C-N): 1497.04 cm^−1^; ν(C-S): 990.26 cm^−1^.

### 3.2. Characterization of the of MnL_2_O_2_ (2) 

After exposure of the yellow solid **1** to atmospheric dioxygen the yellow powder starts to darken and progressively turned into a dark-brown solid.

Analysis calculated for [Mn(S_2_CNEt_2_)_2_O_2_], C_15_H_30_N_3_S_6_O_2_Mn (Mw, 383.48): C, 31.3%; H, 5.3%; S, 33.4%; N, 7.3%. Found: C, 32.6%; H, 5.7%; S, 34.7%; N, 8.2%. ESI-MS: [Mn(S_2_CNEt_2_)_2_O_2_], expected m/z = 382.98, found m/z = 383.06 [M]^+^. FT IR: ν(O-O) 969.32 cm^−1^.

### 3.3. Synthesis and Characterization of MnL_3_ (3)

Method (a). The compound **3** was prepared by dropwise addition of MnCl_2_x4H_2_O (2.5 mmol, 500.8 mg) in 5 mL Milli-Q water to three equivalents of the sodium salt of the ligand L (NaL•3H_2_O) (7.5 mmol, 1.7 g) previously dissolved in 20 mL of Milli-Q water. The preparation was carried out in the open atmosphere and the solution mixed for 30 min. After extraction with 50 mL of chloroform and evaporation of the solvent, a dark violet precipitate (999.5 mg) was obtained and washed with water, ethanol and ether. The compound **3** is soluble in acetonitrile, chloroform and acetone. (Yield, 80%.) Analysis calculated for [Mn(S_2_CNEt_2_)_3_], C_15_H_30_N_3_S_6_Mn (Mw, 499.75): C, 36.1%; H, 6.1%; S, 38.5%; N, 8.4%. Found: C, 36.0%; H, 5.9%; S, 37.6%; N, 8.7%. ESI-MS: [Mn(S_2_CNEt_2_)_3_], expected *m*/*z* = 499.01; found *m*/*z* = 499.40 [M]^+^.

Method (b). 0.19 g of [Mn(PTA)_2_(Cl)_2_(H_2_O)_2_] (0.39 mmol), previously dissolved in 5 mL of Milli-Q water, was added to 10 mL of an aqueous solution containing 0.41 g of NaL•3H_2_O (1.81 mmol). A dark violet precipitate was rapidly formed. The precipitate was filtered under reduced pressure and washed with water and diethyl ether. (Yield, 75% (0.146 g).) Analysis calculated for [Mn(S_2_CNEt_2_)_3_], C_15_H_30_N_3_S_6_Mn (Mw, 499.75): C, 36.1%; H, 6.1%; S, 38.5%; N, 8.4%. Found: C, 36.0%; H, 5.8; %S, 37.3%; N, 8.9%. ESI-MS: [Mn(S_2_CNEt_2_)_3_], expected *m*/*z* = 499.01; found *m*/*z* = 498.60 [M]^+^.

Method (c). A dark solution containing 0.2 g (0.60 mmol) of Mn(III)acetylacetonate [Mn(acac)_3_] in 10 mL of EtOH was slowly added to 20 mL of an aqueous solution containing 0.6g of NaL•3H_2_O (2.80 mmol). The resulting solution was heated at 70 °C for 60 min and the resulting dark violet residue (0.225 g) was then transferred to a test tube and treated with water (5 mL), ethanol (5 mL) and diethyl ether. (Yield > 75%.) Analysis calculated for [Mn(S_2_CNEt_2_)_3_], C_15_H_30_N_3_S_6_Mn (Mw, 499.75): C, 36.1%; H, 6.1%; S, 38.5%; N, 8.4%. Found: C, 35.9%; H, 6.0%; S, 38.6%; N, 8.9%.

### 3.4. Synthesis and Characterization of MnL_2′_ (4)

The compound **4** was prepared by dropwise addition of MnCl_2_x4H_2_O (0.40 mmol, 0.080 g) in 5 mL Milli-Q water to two equivalents of the sodium salt of the ligand L’ (0.80 mmol, 0.240 g) previously dissolved in 5 mL of Milli-Q water. The preparation was carried out under oxygen-free conditions. The immediate formation of a yellow precipitate was observed which, after leaving the reaction for 30 min under magnetic stirring, was filtered and washed with water, still in a controlled atmosphere. (Yield, 70% (0.148 mg).)

FT IR: ν(C–N): 1473.62 cm^−1^; ν(C–S): 996.88 cm^−1^. ESI-MS: [Mn(S_2_CN(CH_2_CH_2_OEt)_2_)_2_], expected *m*/*z* for C_18_H_36_N_2_O_4_S_2_Mn = 527.09; found *m*/*z* = 527.24 [M]^+^.

### 3.5. Characterization of MnL_2′_O_2_ (5)

After exposure of the yellow solid **4** to atmospheric dioxygen, the yellow powder started to darken and progressively turned into a dark brown solid.

Analysis calculated for [Mn(S_2_CN(CH_2_CH_2_OEt)_2_)_2_O_2_], C_18_H_36_N_2_O_6_S_6_Mn (Mw, 559.69): C, 38.6%; H, 6.5%; S, 22.9%; N, 5.0%. Found: C, 38.6%; H, 6.5%; S, 22.9%; N, 6.0%.

FT IR: ν(C–S) 995.80 cm^−1^; ν(C–N) 1484.39 cm^−1^; ν(O–O) 976.62 cm^−1^.

### 3.6. Synthesis and Characterization of MnL’_3_ (6)

The compound **6** was prepared by dropwise addition of MnCl_2_x4H_2_O (0.40 mmol, 0.080 g) in 5 mL Milli-Q water to three equivalents of the sodium salt of the ligand L’ (1.20 mmol, 0.360 g) previously dissolved in 5 mL of Milli-Q water in open atmosphere. The immediate formation of a dark violet precipitate was observed which, after leaving the reaction for 60 min under magnetic stirring, was filtered and washed with water and ether. The compound **6** is soluble in acetonitrile, chloroform and acetone. (Yield, 63% (0.193 g)). Analysis calculated for C_27_H_54_MnN_3_O_6_S_6_ 764.06 (Mw, 764.06): C, 42.4%; H, 7.1%; S, 25.2%; N, 5.5%. Found: C, 42.1%; H, 7.0%; S, 25.2%; N, 5.5%. ESI-MS: [Mn(S_2_CN(CH_2_CH_2_OEt)_2_)_3_], expected *m*/*z* = 763.17; found *m*/*z* = 763.59 [M]^+^. FT IR: ν(C–S) 993.60 cm^−1^; ν(C–N) 1485.97 cm^−1^ (Supplementary Figure S18).

## 4. Conclusions

In our study, we found that the [Mn(II)(S_2_CNEt_2_)_2_] complex can be prepared and is stable only under strictly nitrogen-controlled conditions; and, surprisingly, we found that when the yellow solid [Mn(S_2_CNEt_2_)_2_] compound came into contact with air, the superoxide adduct [Mn(III)(S_2_CNEt_2_)_2_(O_2_)] could be generated, which we assume converts to the tris-substituted complex [Mn(III)(S_2_CNEt)_3_] over time. In their original work, Ciampolini et al. already noted that MnL_2_ instantaneously reacted in the presence of trace amounts of dioxygen [9]. According to our knowledge, these results demonstrate for the first time the involvement of dioxygen in the conversion of the yellow bis-substituted Mn(II) complex to the dark violet tris-Mn(III)complex, which is accelerated in the presence of organic solvents. 

The same reactivity with atmospheric dioxygen was observed with the [Mn(II)(S_2_CN(CH_2_CH_2_OEt)_2_)_2_] complex. The course of the reaction between solid compounds MnL_2_ (**1**) and MnL’_2_ (**4**) and atmospheric dioxygen was monitored over time, and it was found that the ultimate product was always the mixed Mn(II)/Mn(III) oxide hausmannite.

In conclusion, we reported here a new example of small-molecule Mn(II) complexes that efficiently activate dioxygen in the solid state through the formation of an intermediate superoxide adduct. It is known that the activation of dioxygen plays a crucial role in a multitude of chemical and biological processes. Binding of dioxygen to transition metal ions is an essential step in the activation process, and synthetic biomimetic model complexes have been developed with the purpose of revealing the inner mechanism of dioxygen activation in biological systems. 

The potential applications of this intermediate in chemical synthesis and metalloenzymatic reactions remain to be elucidated.

## Figures and Tables

**Table 1:** Effective magnetic susceptibility of the Mn complexes measured using the Evans NMR method (*n* = 3, SD = standard deviation).

Compound	μ_eff_ ± SD
**2**	4.31 ± 0.03
**3**	4.28 ± 0.02
**5**	4.47 ± 0.14
**6**	4.17 ± 0.05
[Mn(III)(acac)_3_]	4.62 ± 0.02

## Data Availability

The data presented in this study are available in supplementary materials.

## Supplementary Materials

The following are available online, Figure S1: IR spectrum of the product 1, Figure S2: ESI(+)-MS spectra of the product 1, Figure S3: XRD pattern of the newly form brown product after exposure of the product 1 to air, Figure S4: ESI(+)-MS spectrum of the dark product collected 24 hours after exposure of 1 to atmospheric oxygen, Figure S5: UV-Vis spectra of the dark product collected 24 hours after exposure of 1 to atmospheric oxygen, Figure S6: FT-IR spectrum of the dark product collected 24 hours after exposure of 1 to atmospheric dioxygen, Figure S7: FT-IR spectrum of the brown-ochre sample collected after 10 days of exposition of 2 to atmospheric dioxygen, Figure S8: XRD pattern of brown-ochre sample collected after 10 days of exposition of 2 to atmospheric dioxygen, Figure S9: ESI(+)-MS spectra of the product 3, Figure S10: The electronic spectrum of 3, Figure S11: Cyclic voltammetry of the complex 3 in 0.1 M TBACl/DCM, Figure S12: FT-IR spectrum of the product 4, Figure S13: FT-IR spectrum of dark-brown solid product after exposition of the product 4 to atmospheric dioxygen, Figure S14: ESI(+)-MS spectrum of dark-brown solid product after exposition of the product 4 to atmospheric dioxygen, Figure S15: FT-IR spectrum of the yellow-ocher powder separated after treatment with ether of the product 5, Figure S16: FT-IR spectrum of the dark-violet powder separated after treatment with ether of the product 5, Figure S17: Example of ^1^H NMR spectrum obtained for the determination of effective magnetic moment of 5 using the Evans method, Figure S18: FT-IR spectrum of the product 6.

## Sample Availability

Samples of the compounds **3** and **6** are available from the authors.
