# Supplementary material for: Synthesis and Characterization of Manganese Dithiocarbamate Complexes: New Evidence of Dioxygen Activation
Source: Molecules. 2021 Sep 30;26(19):5954. doi: 10.3390/molecules26195954 (PMC8512220; doi:10.3390/molecules26195954)
Supplement: Supplementary file 1 [file molecules-26-05954-s001.zip › molecules-1406580-supplementary.pdf]

molecules

MDPI

Supporting Information for:

Synthesis and Characterization of Manganese Dithiocarbamate complexes: new evidence of di-oxygen activation

Petra Martini[1], Alessandra Boschi[2]*, Lorenza Marvelli[2], Licia Uccelli[1], Stefano Carli[2], Giuseppe Cruciani[3], Erika Marzola[2], Anna Fantinati[2], Juan Esposito[4] and Adriano Duatti[2]

[1] Department of Translational Medicine, via Fossato di Mortara, 70 c/o viale Eliporto - 44121 Ferrara, Italy; petra.martini@unife.it, (P.M.); licia.uccelli@unife.it, (L.U.);

[2] Department of Chemical, Pharmaceutical and Agricultural Sciences, Via L. Borsari, 46-44121 Ferrara, Italy; alessandra.boschi@unife.it, (A.B.); lorenza.marvelli@unife.it, (L.M.); dta@unife.it, (A.D.); stefano.carli@unife.it, (S.C.); erika.marzola@unife.it, (E.M.); anna.fantinati@unife.it, (A.F.);

[3] Department of Physics and Earth Sciences, Via Saragat, 1-44122 Ferrara, Italy; giuseppe.cruciani@unife.it; (G.C.);

[4] Legnaro National Laboratories, Italian National Institute for Nuclear Physics (LNL-INFN), Viale dell'Università, 2, 35020 Legnaro (PD), Italy; juan.esposito@lnl.infn.it; (J.E.);

* Correspondence: (A.B.) alessandra.boschi@unife.it, Tel.+39 0532 455354.

2.1 Synthesis and characterization of MnL_2 (1), (L = diethyldithiocarbamate).

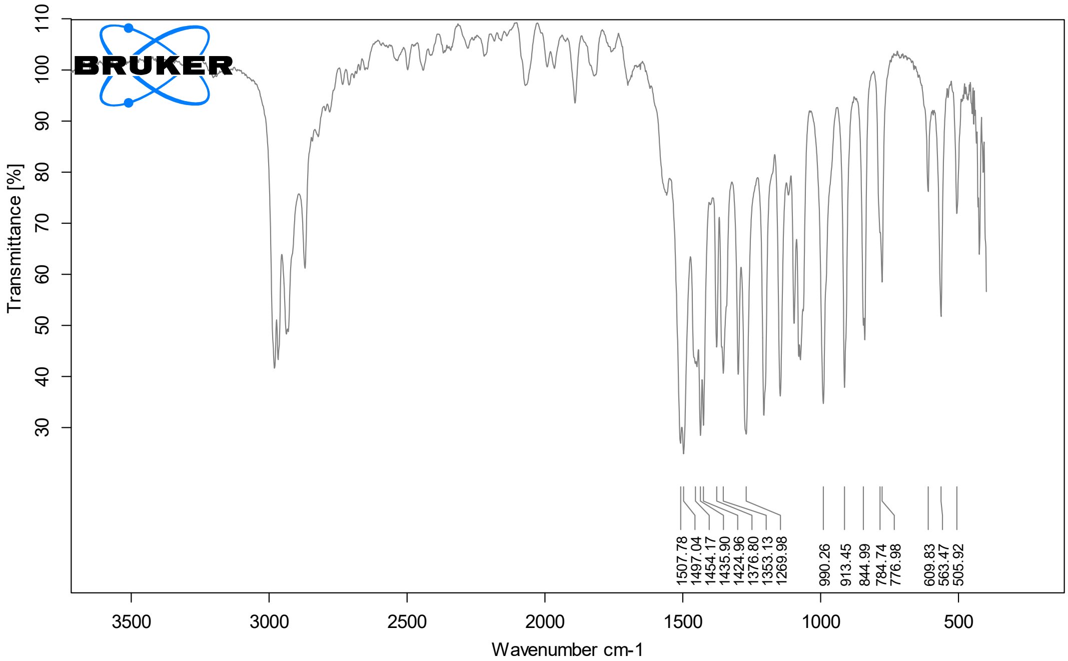

Figure S1. IR spectrum of the product **1** obtained under inert condition

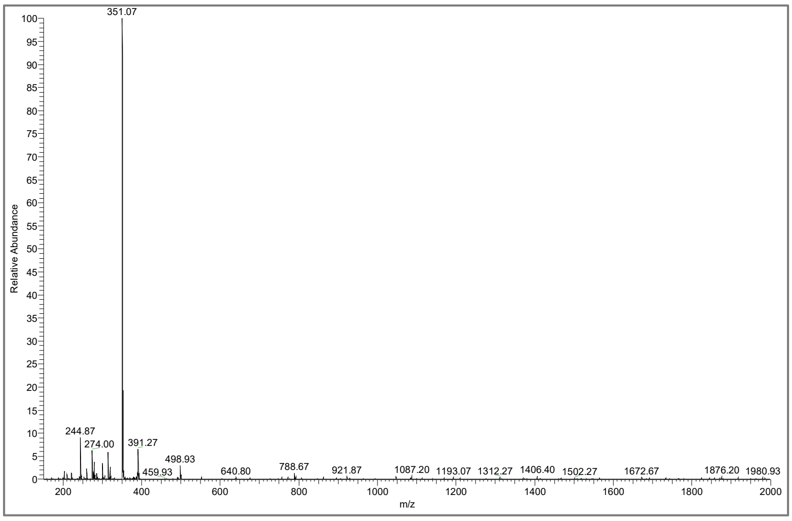

Figure S2. ESI(+)-MS spectra of the product **1** obtained in CH_3CN, average source voltage 4.5 kV. Expected m/z for **1**, $C_{10}H_{20}NS_2Mn$ = 351.00, found m/z = 351.07 $[M]^+$.

2.2. *Stability studies of MnL_2 (1) after exposition to atmospheric dioxygen*

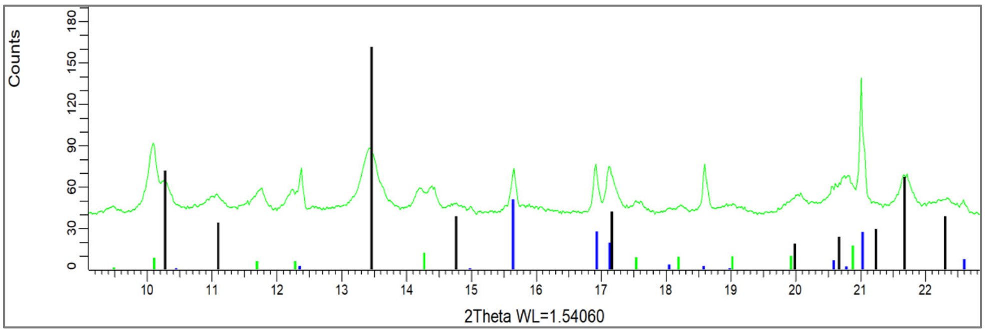

Figure S3. XRD pattern of the newly form brown product after exposure of the product **1** to air. The lime bars show the presence of the bis-substituted complex MnL_2 (**1**), the black ones the presence of an unidentified phase and the blue ones of the ligand NaL.

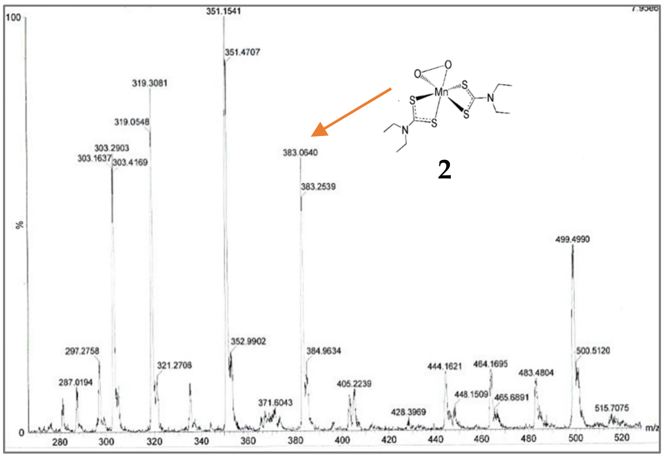

Figure S4. ESI(+)-MS spectrum of the dark product collected 24 hours after exposure of **1** to atmospheric oxygen

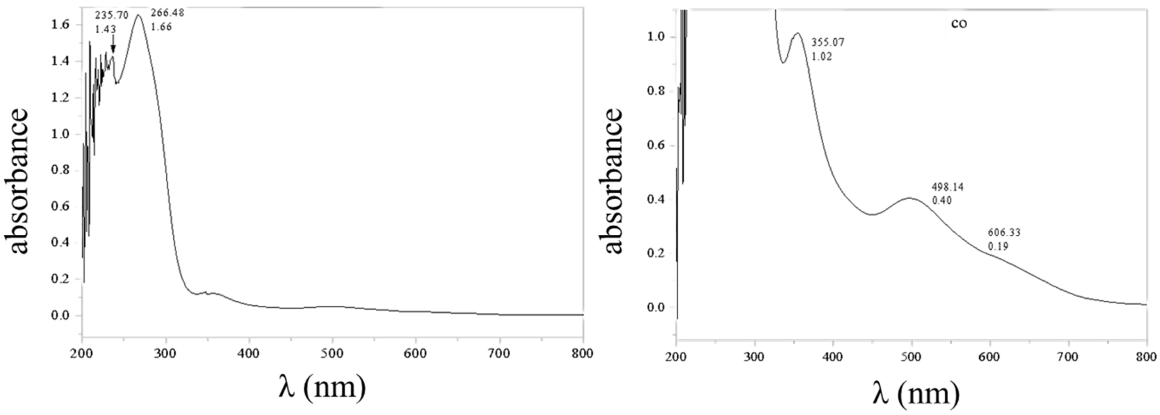

a **b**

Figure S5. UV-Vis spectra of the dark product collected 24 hours after exposure of **1** to atmospheric oxygen obtained in chloroform, dilute sample **(a)**, concentrated sample **(b)**.

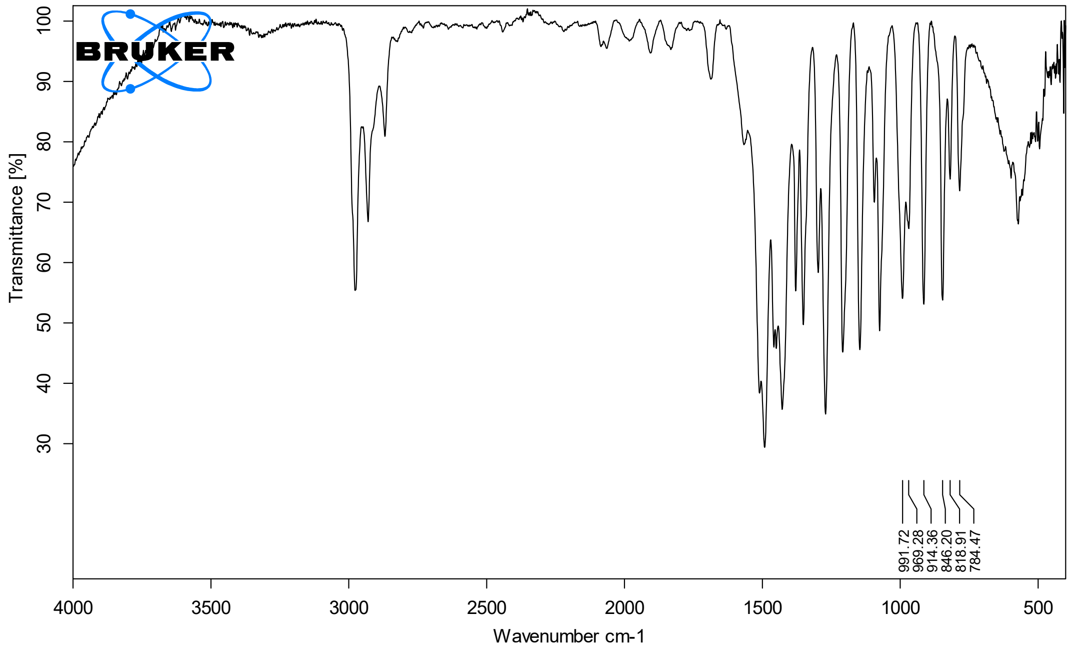

Figure S6. FT-IR spectrum of the dark product collected 24 hours after exposure of **1** to atmospheric dioxygen

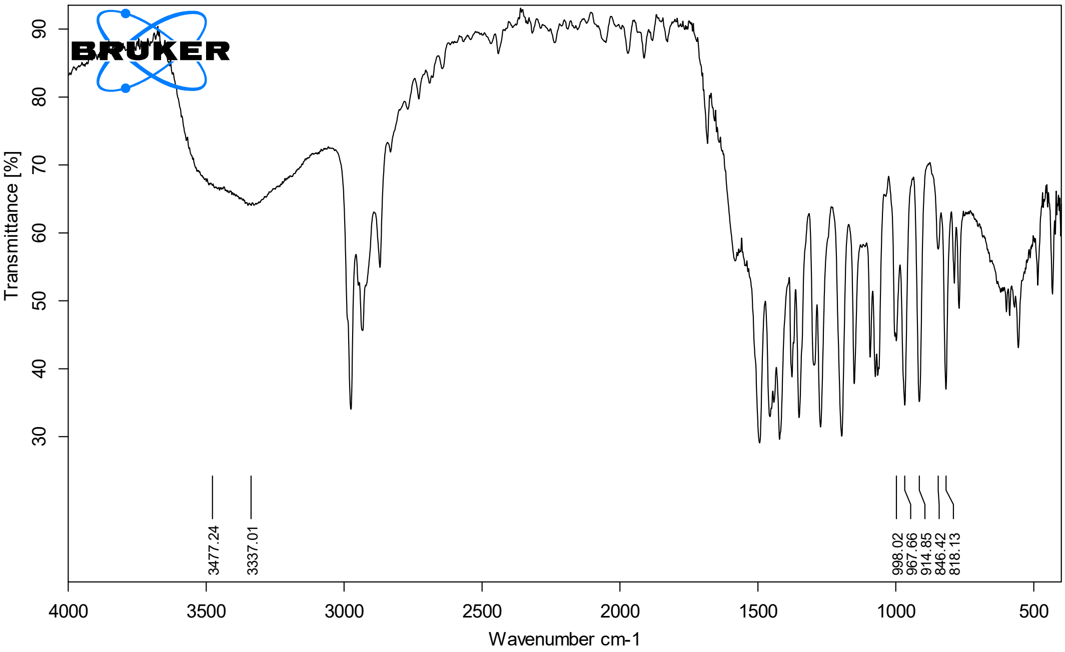

Figure S7. FT-IR spectrum of the brown-ochre sample collected after 10 days of exposition of **2** to atmospheric dioxygen.

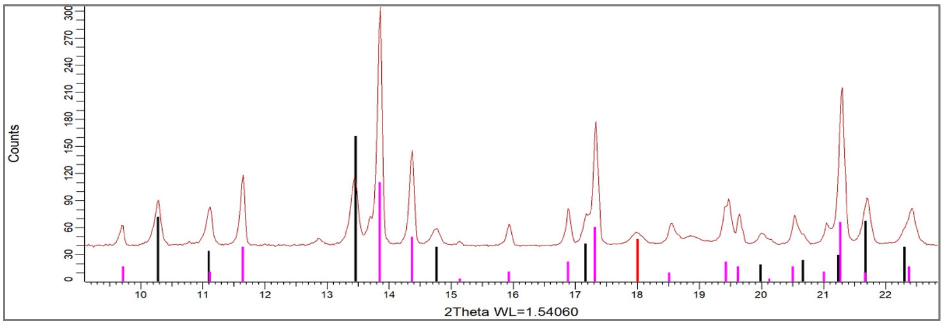

Figure S8. XRD pattern of brown-ochre sample collected after 10 days of exposition of **2** to atmospheric dioxygen. The pink bars show the presence of $C_{10}H_{20}N_2S_4$ (Disulfiram), the black ones the presence of the unidentified phase and the red one of *syn*-Mn_3O_4.

2.3. Synthesis and characterization of [MnL_3] (3), (L = diethyldithiocarbamate)

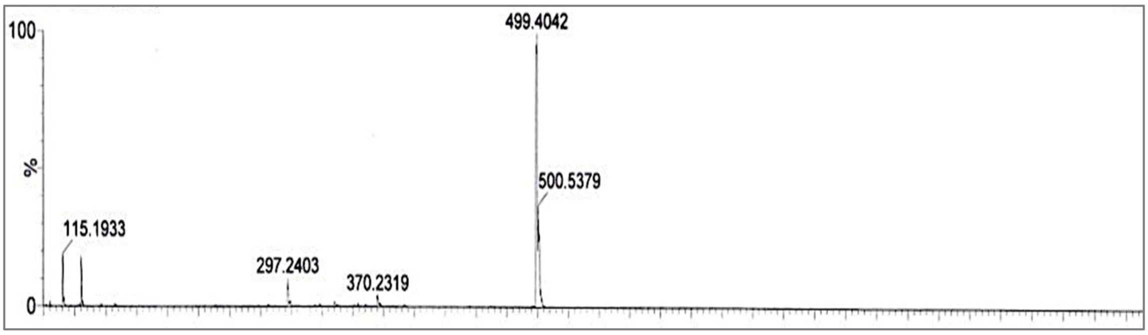

Figure S9. ESI(+)-MS spectra of the product **3**

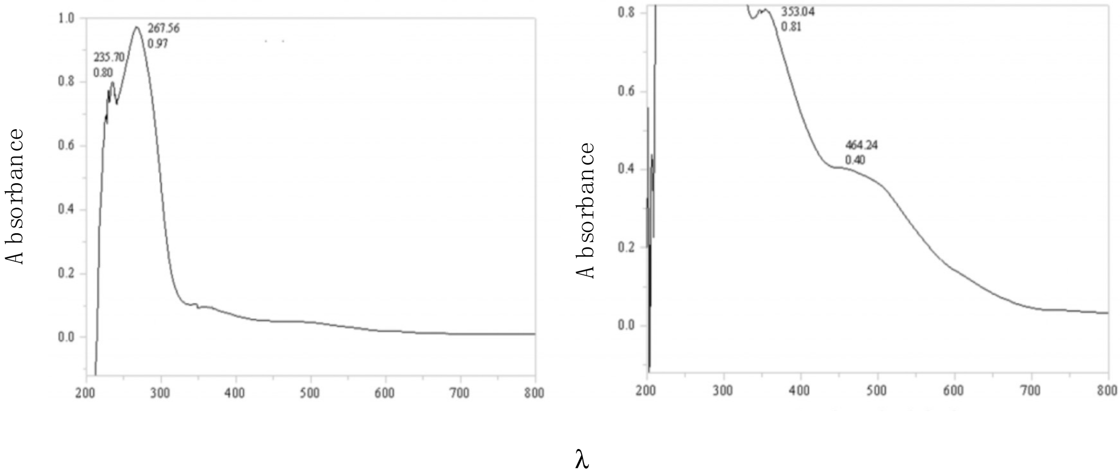

Figure S10. The electronic spectrum of **3**.

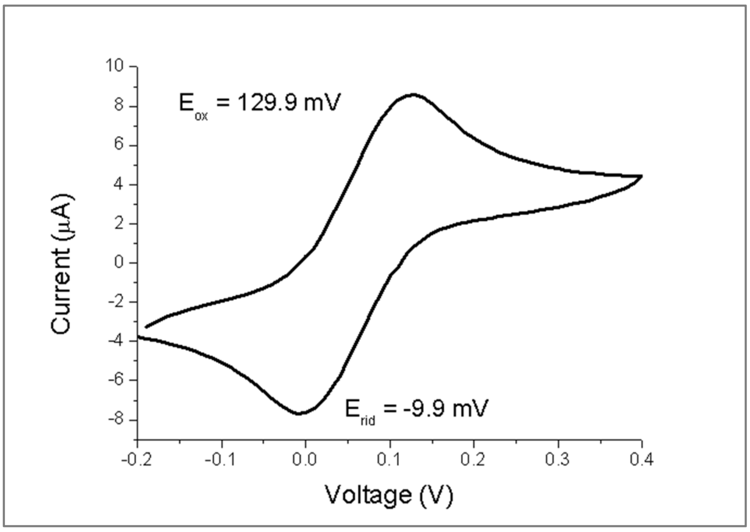

Figure S11. Cyclic voltammetry of the complex **3** in 0.1 M TBACl/DCM: WE glassy carbon, CE Pt wire, pseudo-RE Ag wire, scan rate 100 mV/s.

2.4. Synthesis, characterization of [Mn(II)(($S_2CN(CH_2CH_2OEt)_2)_2$] (4) L′ = bis(N-ethoxyethyl)dithiocarbamate and stability studies after exposition to atmospheric dioxygen

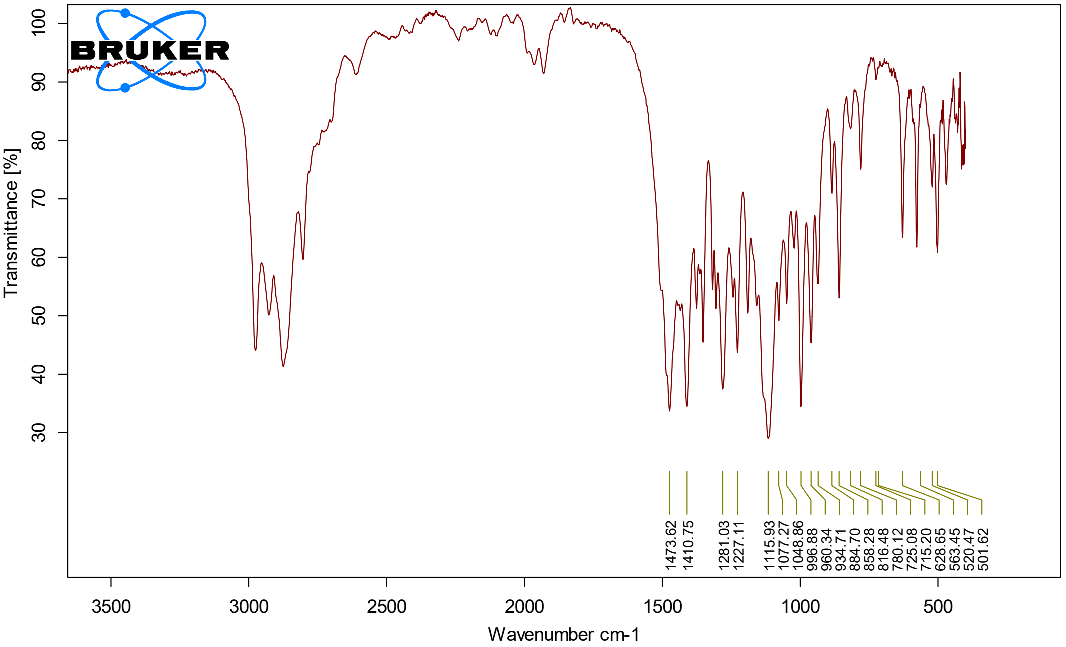

Figure S12. FT-IR spectrum of the product **4**.

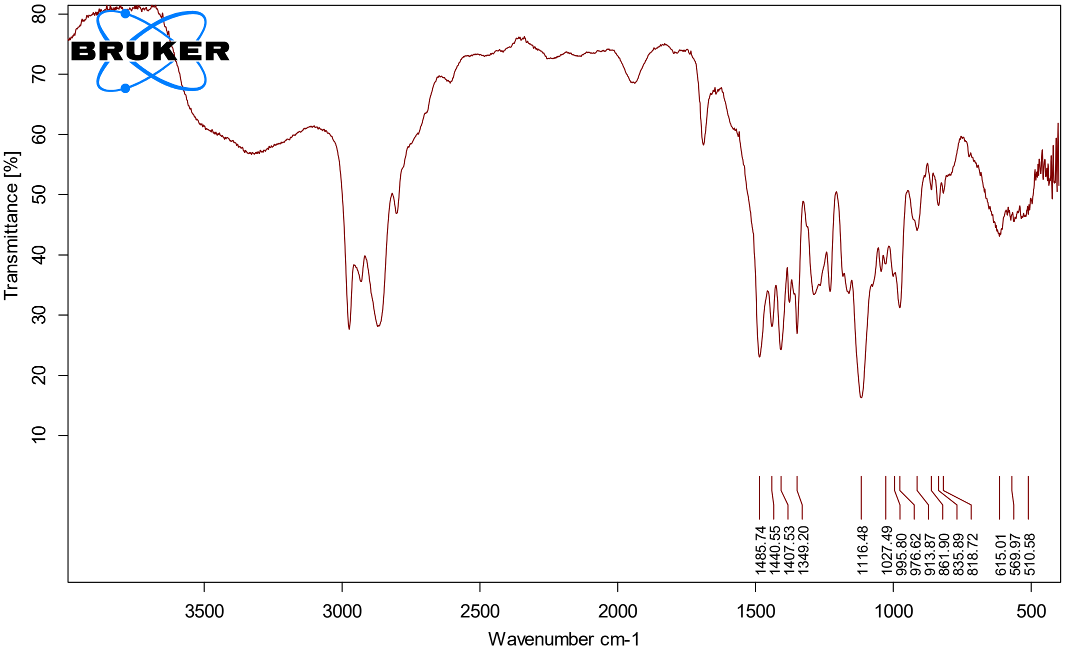

Figure S13. FT-IR spectrum of dark-brown solid product after exposition of the product **4** to atmospheric dioxygen.

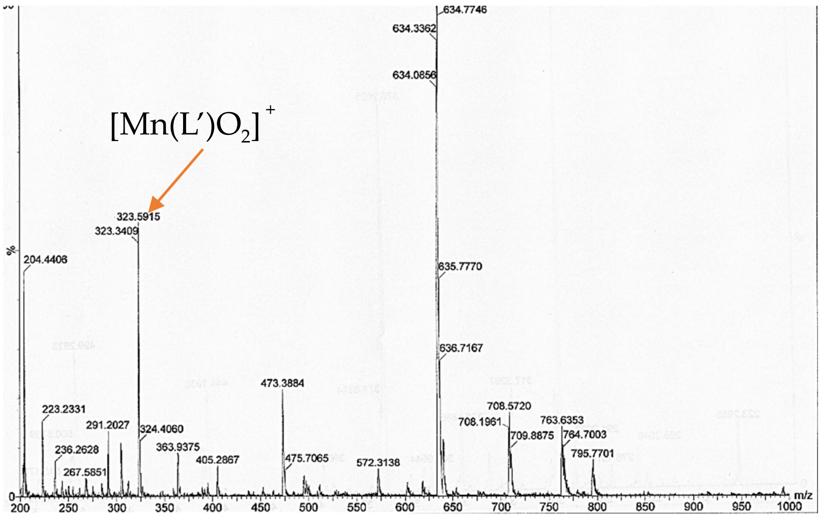

Figure S14. ESI(+)-MS spectrum of dark-brown solid product after exposition of the product **4** to atmospheric dioxygen.

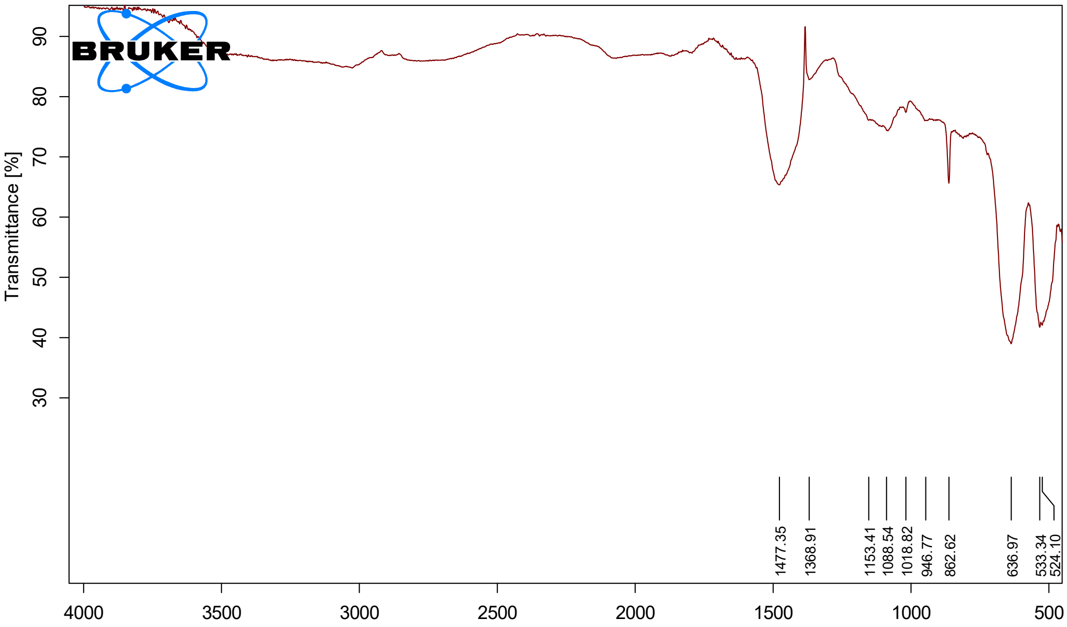

Figure S15. FT-IR spectrum of the yellow-ocher powder separated after treatment with ether of the product **5**.

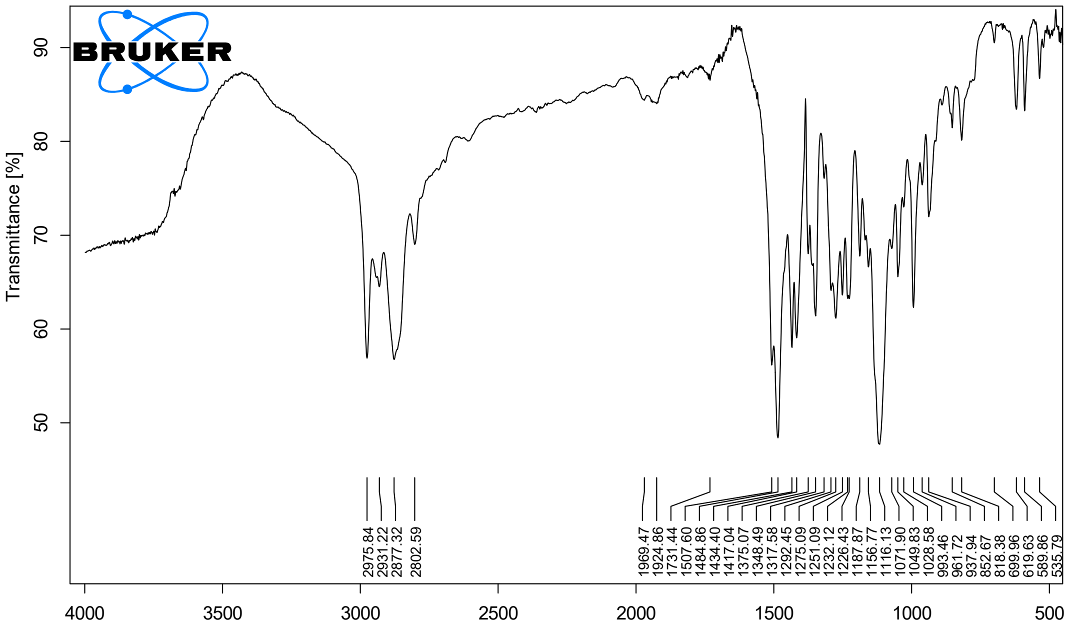

Figure S16. FT-IR spectrum of the dark-violet powder separated after treatment with ether of the product **5**.

Characterization of dark-violet powder separated after treatment with ether of the product **5** FT-IR ν(C-S) 993,41 cm^{-1} ; ν (C-N) 1484,95 cm^{-1}. AE for $C_{27}H_{54}MnN_3O_6S_6$ 764.06 (Mw, 764.06): C, 42.4%; H, 7.1%; S, 25.2%; N, 5.5%. Found: C, 42.0%; H, 7.1%; S, 25.2%; N, 5.5%. ESI-MS: $[Mn((S_2CN(CH_2CH_2OEt)_2)_3]$, expected m/z = 763.17, found m/z = 763.59 $[M]^+$.

2.5 Magnetic susceptibility

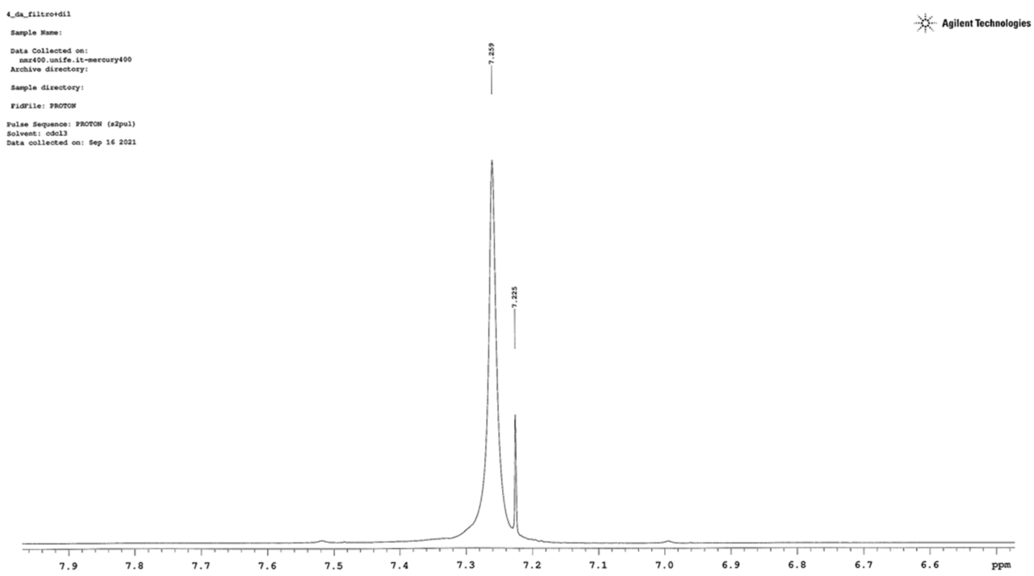

Figure S17. Example of ^{1}H NMR spectrum obtained for the determination of effective magnetic moment of **5** using the Evans method

3.6. Synthesis and characterization of MnL′₃ (6)

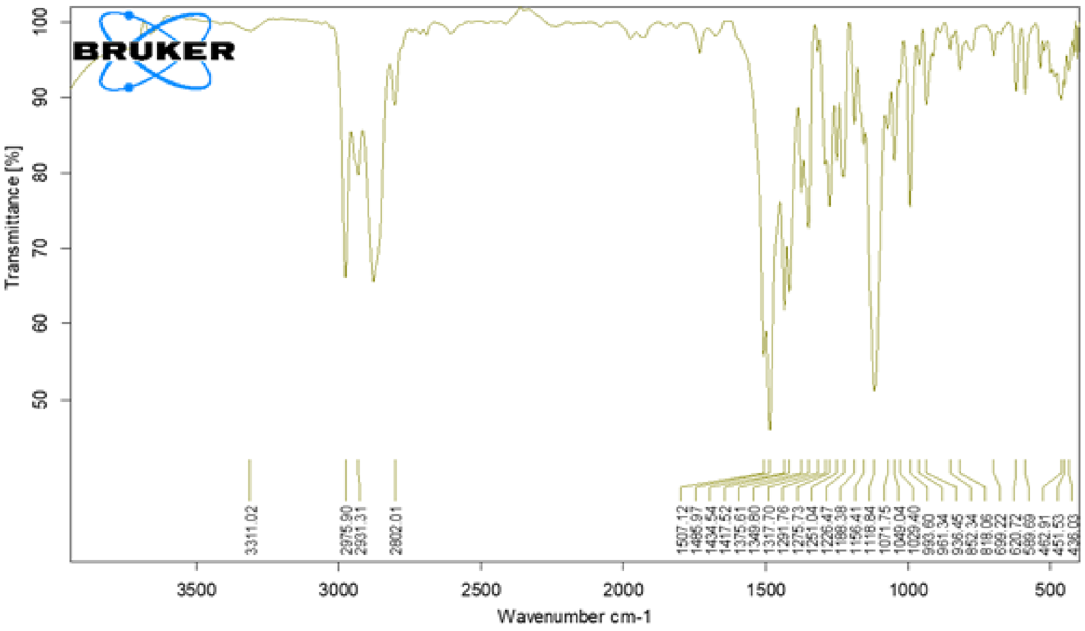

Figure S18. FT-IR spectrum of the product **6.**
